# The Association between the Bisphenols Residues in Amniotic Fluid and Fetal Abnormalities in Polish Pregnant Women—Its Potential Clinical Application

**DOI:** 10.3390/ijms24010730

**Published:** 2023-01-01

**Authors:** Tomasz Tuzimski, Szymon Szubartowski, Aleksandra Stupak, Wojciech Kwaśniewski, Małgorzata Szultka-Młyńska, Anna Kwaśniewska, Bogusław Buszewski

**Affiliations:** 1 Department of Physical Chemistry, Medical University of Lublin, 20-093 Lublin, Poland; 2 Doctoral School of Medical University of Lublin, Medical University of Lublin, 20-093 Lublin, Poland; 3 Chair and Department of Obstetrics and Pathology of Pregnancy, Independent Public Clinical Hospital No. 1 in Lublin, Medical University of Lublin, 20-081 Lublin, Poland; 4 Department of Gynaecology and Oncology Gynaecology, Independent Public Clinical Hospital No. 1 in Lublin, Medical University of Lublin, 20-081 Lublin, Poland; 5 Department of Environmental Chemistry and Bioanalytics, Faculty of Chemistry, Nicolaus Copernicus University, 87-100 Torun, Poland

**Keywords:** amniotic fluid (AF), bisphenol A (BPA), BPA analogues (F, S, AF), bisphenol A diglicydyl ether (BADGE) and analogues, fetal chromosomal abnormalities, trisomy

## Abstract

The present study aimed to investigate the relationship between the concentrations of bisphenols residues in the amniotic fluid (AF) samples collected during amniocentesis and fetal chromosomal abnormalities in pregnant women. A total of 33 pregnant Polish women aged between 24 and 44 years, and screened to detect high risk for chromosomal defects in the first trimester, were included in this study. Samples were collected from these patients during routine diagnostic and treatment procedures at mid-gestation. The concentrations of various bisphenols residues in the samples were determined by liquid chromatography coupled with triple quadrupole tandem mass spectrometry (LC-ESI-QqQ-MS/MS). Residues of eight analytes (BPS, BPF, BPA, BPAF, BADGE, BADGE•2H_2_O, BADGE•H_2_O•HCl and BADGE•2HCl) were detected in amniotic fluid samples in the range 0.69 ng/mL to 3.38 ng/mL. Fetuses with chromosomal abnormalities showed a slightly higher frequency of occurrence of selected bisphenols residues in the AF samples collected between 15–26 weeks of pregnancies. Finally, the proposed method was applied in the simultaneous determination of several endocrine-disrupting chemicals from bisphenol group in 33 human AF samples. BADGE•H2O•HCl has been identified in the AF samples taken from women older than average in the examined group. The number of detected compounds has been significant for the following analytes: BPS, BPAF, BADGE•H_2_O•HCl and BADGE. The proposed method may be an attractive alternative for application in large-scale human biomonitoring studies.

## 1. Introduction

Prenatal diagnosis is a very important element of genetic diagnosis. Its development was made possible by achievements in several areas. All pregnant women are offered a first-trimester ultrasound scan according to International Society of Ultrasound in Obstetrics and Gynecology (ISUOG) guidelines [1,2]. Screening strategies are based on individual risk calculated from maternal age and nuchal translucency measurement and/or maternal serum markers and/or other ultrasound markers in the first trimester (defined by the conventional crown–rump length (CRL) range of 45–84 mm) [3]. The abnormal result allows the selection of a subgroup of patients in whom further tests for aneuploids syndromes or neural tube defects should be performed [4]. Prenatal diagnosis methods can be divided into two main categories: fetal tissue analysis (invasive and non-invasive) and fetal imaging. Fetal tissue analysis includes amniocentesis, chorionic villus sampling, cordocentesis and genetic preimplantation diagnostics. Non-invasive prenatal screening for trisomy 21 (Down syndrome), trisomy 18 (Edwards syndrome), trisomy 13 (Patau syndrome), and selected sex chromosome aneuploids can be performed using next-generation sequencing of cell-free DNA (cfDNA) in the maternal circulation [5]. Ultrasound and nuclear magnetic resonance are methods of imaging the fetus [4]. All pregnant women are informed of the benefits and limitations of undergoing prenatal screening for fetal aneuploids and provide prior informed consent. Amniocentesis is an invasive method (collection of amniotic fluid) and represents the most accurate diagnostic method. The most common diagnostic indications for obtaining amniotic fluid are prenatal genetic studies. Other indications include the evaluation of the fetus for infection, the degree of hemolytic anemia, blood or platelet type, hemoglobinopathy, and neural tube defects. Amniocentesis should be performed at or beyond 15  +  0 completed weeks of gestation, starting from the date of the last period and confirmed by US scan in the first trimester measurement of CRL of the fetus [1]. A 20–22-G needle should be inserted transabdominally under continuous ultrasound guidance. Needle entry through the placental cord insertion site must be avoided and, if technically feasible, the avoidance of the placenta is preferable. Approximately 20–30 mL of amniotic fluid containing living cells (amniocytes) is collected. Amniocytes are cultured for approximately 7 days, and then standard cytogenetics tests are performed (traditional karyotype analysis). In addition, cells can be biochemically tested or DNA can be isolated from them for testing for diseases, for which mutation detection methods have been developed (aCGH microarray). Cytogenetic test results are available after 10–12 days.

There are several indications for amniocentesis [1,2,3] such as the assessment of risk for chromosomal abnormalities carried out by a combination of maternal age, fetal nuchal translucency (NT) thickness, and maternal serum free β-human chorionic gonadotrophin (hCG) and pregnancy-associated plasma protein-A (PAPP-A) at 11 to 13 + 6 weeks, a risk of 1 in 300 or more is generally considered to be high; and screening for free circulating DNA in maternal plasma, showing aneuploidy.

The safety and accuracy of amniocentesis has been assessed in many studies. The risk of maternal complications in pregnancy is very small. For women undergoing amniocentesis, the additional risk of fetal loss in comparison with controls has been reported to vary from 0.1% to 1%. The risk of membrane rupture after amniocentesis is 1–2%. Fetal injury and serious maternal complications are rare events. The transient leakage of amniotic fluid occurs in 1% of patients; infections are extremely rare [1].

Chromosomal aberrations are responsible for the significant course of genetically determined diseases, occurring in an estimated one in 150 live births. They are the main cause of intellectual disability and pregnancy loss, as well as the cause of congenital malformations. Chromosome aberrations are detected in 50% of pregnancies lost in the first trimester and approximately 20% of spontaneous abortions in the second trimester. They are therefore a significant cause of mortality. Aneuploidy is a common finding in pregnancy, with a wide spectrum of medical consequences of benign to lethal [6]. The most frequent autosomal trisomy at birth is trisomy 21 (Down syndrome). Its birth prevalence, in the absence of prenatal diagnosis and therapeutic abortion, of 1 to 2 per 1000 in developed countries. Trisomies 18 and 13 (Edwards and Pataua syndromes), have, respectively, about one-tenth, and one-twentieth the birth prevalence. Other autosomal trisomies are associated with early pregnancy loss. Monosomy X (Turner syndrome) is also common early in pregnancy, and mosaic cases can survive to birth. Other sex chromosome abnormalities that are relatively benign are also commonly encountered in livebirths. Triploidy is common in early pregnancy, but the birth prevalence is very low.

The diagnostic value of amniotic fluid is broad and has not yet been fully explored for prenatal diagnosis of pregnancies at risk from xenobiotics, environmental exposures and for the elucidation of mechanisms underlying important public health challenges, including on preterm birth. Despite significant progress in diagnosis, preterm delivery rates remain high. The assessment of human fetal exposure to chemicals is key to fully understand developmental toxicity. In published work by Suliburska et al., the fetuses with chromosomal abnormalities more frequently showed lower manganane concentration in the AF samples collected in the second trimester as compared to those with normal karyotype [7].

Nowadays, a class of substances named endocrine-disrupting chemicals (EDCs) has been gaining the attention of the scientific community due to their potentially harmful effects on human health. Endocrine-disrupting chemicals are present in a wide range of products, mainly in those of daily use, like personal care products, pharmaceuticals and processed food. Endocrine-disrupting chemicals are a group of emerging contaminants that alters the function of the endocrine system, causing possible adverse health effects [8]. Bisphenol A (BPA) is the most representative endocrine disruptor among the bisphenols analogues, and it is one of the most used products in contact materials around the world [9,10,11]. BPA is an additive for the production of phenol resins, polyacrylates, polyesters, epoxy resins and polycarbonate plastics [12,13]. As such, human exposure to BPA occurs through several consumption products such as food and beverage packaging, adhesives, toys, water pipes, drinking containers, eyeglass lenses, sports safety equipment, medical devices, thermal receipts and electronics [13,14]. 

The capability of BPA to migrate from plastic bottles into drinking water is of particular concern, as it might represent a direct route to human contamination [15]. The American Environmental Protection Agency established that BPA appeared in the blood and urine of 95% of people tested [16]. In vitro and in vivo BPA studies have shown considerable damage in male and female reproductive systems, carcinogenesis, and mutagenic and genotoxicity capabilities [17,18,19,20]. In another study, authors showed that tricyclic bisphenols elicit antagonistic activity against both ERα and Erβ [21,22]. Bisphenol analogues have similar physicochemical properties to BPA, which make them suitable candidates for its replacement in several industrial applications. Unfortunately, this similarity also leads to harmful toxicological profiles [23,24]. Kitamura, in a published paper, demonstrated that BPA, BPB and BPS were potent anti-androgens compounds [25]. In another study, Castro et al. [26] obtained in vivo evidence of the potential adverse effects of BPF and BPS in the developing brain of mammals. In another paper, Rosenmai et al. [27] noticed that the main effect resulting from exposure to bisphenols was endocrine interference; while BPS showed low estrogenic and anti-androgenic activity, the other bisphenol analogues had toxicological behavior similar to BPA [28]. The evidence of additional harmful effects includes genotoxicity, carcinogenicity and DNA damage [29], oxidative stress [30,31] and cytotoxicity in human peripheral blood mononuclear cells [32].

So far, only a few research results have been published on the identification of bisphenols (especially BPA) in the amniotic fluid samples [33,34,35]. BPA was present in serum and follicular fluid at ∼1–2 ng/mL, as well as in fetal serum and full-term amniotic fluid, confirming passage through the placenta [36]. Surprisingly, an ∼5-fold higher concentration, 8.3 ± 8.7 ng/mL, was revealed in amniotic fluid at 15–18 weeks gestation, compared with other fluids [36]. Pinney et al. described and characterized human fetal BPA exposure by measuring BPA concentration in second trimester amniotic fluid samples and to study its relationship with birth weight (BW) in full term infants [37]. Their study indicate that the developing fetus is exposed to BPA as it passes through the placenta and into the AF during gestation. In the population of AF samples from healthy second trimester term singleton pregnancies, BPA concentrations in the range of 0.40–2.0 ng/mL were associated with lower BW [37]. The development of a novel assay for total BPA measurement in body fluids through the derivatization of PFB-BPA permitted a sensitive and specific method for the detection of BPA exposure to the fetus [37]. 

In another study, fetal catheterizations were conducted in pregnant sheep (*n* = 6) at mid-pregnancy and injected with either a single dose of BPS (*n* = 3, 0.5 mg/kg, s.c.), or a combination of BPS, BPF and BPA (*n* = 3, 0.5 mg/kg for each chemical, s.c.) [9]. Maternal and fetal blood and urine and amniotic fluid were collected over 72 h and analyzed for bisphenols by HPLC-MS/MS [9]. The authors of this study observed significant differences in half-life, maximum concentration, and total body clearance in maternal circulation among bisphenols [9]. Longer half-lives were observed in fetal vs. maternal circulation for all bisphenols. Fetal toxicokinetics differed among bisphenols with BPS having the longest fetal half-life. All bisphenols reached basal levels at 48 h in maternal plasma, but were still detectable in amniotic fluid, fetal urine, and fetal plasma at 72 h [9].

In the review described by Vrachnis et al., it recommended that exposure to this endocrine disruptor should be as limited as possible during this time period [38]. The actual cut-off value of BPA exposure that could predict abnormalities of fetal growth remains as yet unknown. Moreover, given that combined exposure to dietary and non-dietary sources cannot be considered safe, it certainly seems reasonable to question whether usual environmental exposure during early pregnancy should be regarded as potentially hazardous [38]. In view of the conflicting results of the included studies, more studies are needed in the field to fully elucidate the role of BPA in fetal growth. Although recent studies have contributed significantly to our knowledge about BPA, more are required focusing on its sex-specific effects and, importantly, examining during which gestational period the fetus is most susceptible [38].

New efficient and sensitive analytical methods based on gas chromatography (GC) and liquid chromatography (LC), coupled to mass spectrometry (MS) or tandem mass spectrometry (MS/MS), have been proposed for the trace analysis of BPA and analogous in different matrices [39,40,41]. In our previous publication, a low-cost, specificity, and sensitive method was proposed for the identification and quantitative analysis of 11 bisphenols in amniotic fluid samples collected during an amniocentesis [34]. The published procedure has been combined the advantages of SPE as extraction techniques with HPLC-FLD for the identification and quantification of analytes in human amniotic fluid samples, which have been collected from patients with an indication for amniocentesis in the 15–26th week of pregnancy [34]. 

This publication attempts to link the frequency of bisphenols and their concentrations in the amniotic fluid collected during amniocentesis, and an attempt was made to correlate of frequency/concentrations of bisphenols with chromosomal aberrations.

## 2. Results

### 2.1. LC-ESI-QqQ-MS/MS Conditions

**Figure 1:**
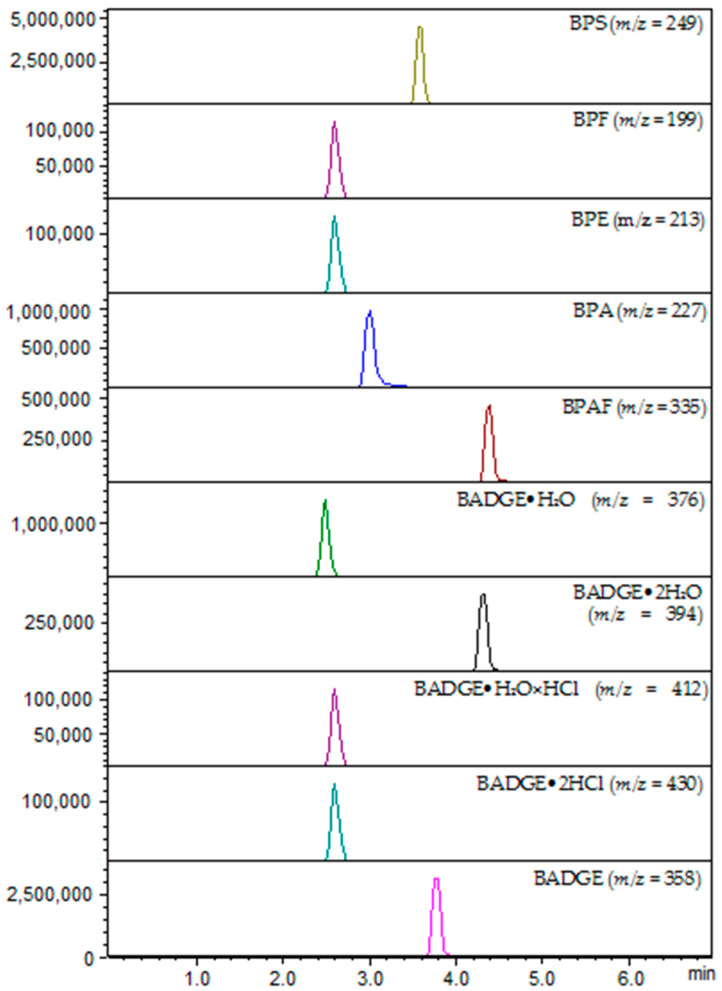
Representative MRM chromatograms of 10 studied bisphenols. BPS (*m*/*z* = 249), BPF (*m*/*z* = 199), BPE (*m*/*z* = 213), BPA (*m*/*z* = 227), BPAF (*m*/*z* = 335), BADGE•2H_2_O (*m*/*z* = 394), BADGE•H_2_O (*m*/*z* = 376), BADGE•H_2_O×HCl (*m*/*z* = 412), BADGE•2HCl (*m*/*z* = 430), BADGE (*m*/*z* = 358).

MS/MS experiments were performed in the QqQ mass spectrometer (8050 Shimadzu (Kyoto, Japan)) equipped with an electrospray ionization (ESI) source. After determining the best conditions for isolating the precursor on (analyte proton adduct), full scan MS/MS mode was used to record product ions from the standard solution of each target compound. The fragmentation amplitude and isolation width for each analyte were manually optimized to increase the method’s selectivity and sensitivity and to select the most intense and characteristic fragmentation ions for qualitative analysis and one of the highest intensity for quantitative analysis. Exemplarily, MRM chromatograms of the studied bisphenols are presented in Figure 1.

**Figure 2:**
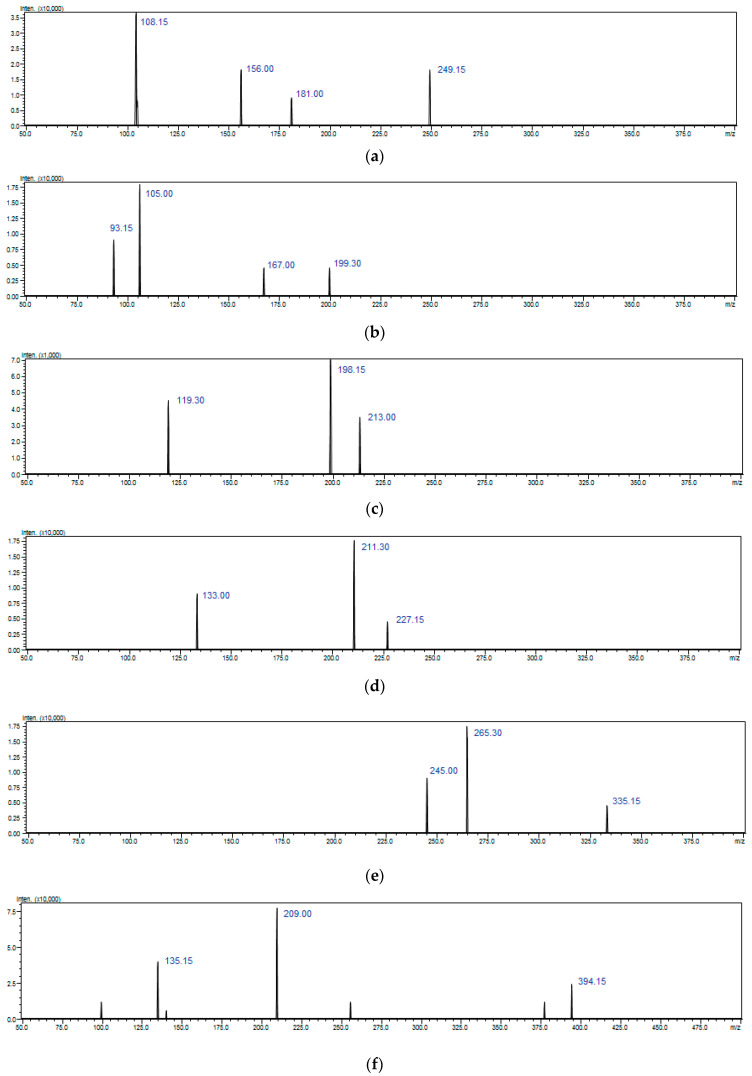

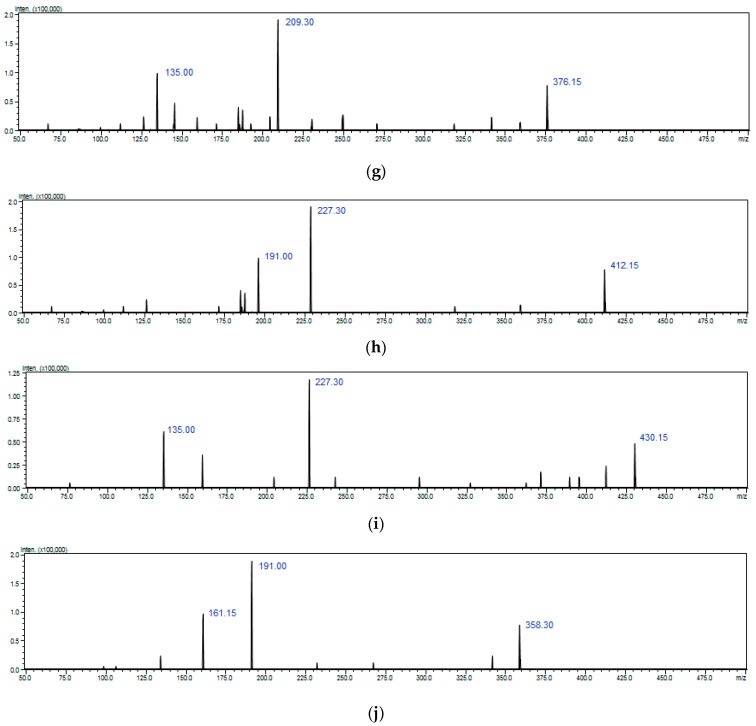
QqQ-ESI-MS/MS spectra of (**a**) BPS (*m*/*z* = 249), (**b**) BPF (*m*/*z* = 199), (**c**) BPE (*m*/*z* = 213), (**d**) BPA (*m*/*z* = 227), (**e**) BPAF (*m*/*z* = 335), (**f**) BADGE•2H_2_O (*m*/*z* = 394), (**g**) BADGE•H_2_O (*m*/*z* = 376), (**h**) BADGE•H_2_O•HCl (*m*/*z* = 412), (**i**) BADGE•2HCl (*m*/*z* = 430), (**j**) BADGE (*m*/*z* = 358).

The LC-ESI-QqQ-MS/MS method was applied for the determination and identification of studied bisphenols (BPSs). BPS (*m*/*z* = 249), BPF (*m*/*z* = 199), BPE (*m*/*z* = 213), BPA (*m*/*z* = 227), BPAF (*m*/*z* = 335) showed intense [M−H]^−^ ions in the negative ionization mode of the analysis (ESI^−^). On the other hand, BADGE•2H_2_O (*m*/*z* = 394), BADGE•H_2_O (*m*/*z* = 376), BADGE•H_2_O•HCl (*m*/*z* = 412), BADGE•2HCl (*m*/*z* = 430), BADGE (*m*/*z* = 358) showed intense [M+NH_4_]^+^ ions in the positive ionization mode of the analysis (ESI^+^). The identified BPSs were further characterized based on the MS/MS fragmentation patterns (Figure 2).

### 2.2. Liquid Chromatography-Tandem Mass Spectrometry (LC-MS/MS)

Liquid chromatography and mass spectrometry was used to identify the selected bisphenols (BPSs) in the amniotic fluid samples. The ESI mass spectra and the MS/MS product ion spectra of BPSs were obtained by a direct infusion of the analyte solutions via a tee connection between the LC and the mass spectrometer. After the MS operating parameters were optimized, the subsequent methods using single ion monitoring (SIM) and multiple-reaction monitoring (MRM) to analyze BPSs were generated and compared. In this study, ESI was used to ionize BPSs in negative mode (for BPS, BPF, BPE, BPA, BPAF) using MeOH: water as mobile phase or in positive mode (for BADGE•2H_2_O, BADGE•H_2_O, BADGE•H_2_O•HCl, BADGE•2HCl, BADGE) using ammonium formate and methanol.

BPS contains two phenolic hydroxyl groups with one sulfinyl on each side, and has a higher heat stability than BPA. Hence, in its MS/MS fragmentation spectrum, the ion at *m*/*z* 156 was generated due to the breakage of two carbon-sulfur bonds and was assigned as [M-H-C_6_H_5_O]^−^. Product ion at *m*/*z* 108 was formed by losing one sulfinyl and one phenol. In the MS/MS spectrum of [M−H]^−^ of BPF, product ions at *m*/*z* 93 and 105 were assigned to [M-H-C_7_H_6_O]^−^ and [M-H-C_6_H_6_O]^−^ respectively. In the MS/MS spectrum of [M-H]^−^ of BPA, product ions at *m*/*z* 211 and 133 were assigned to [M-H-CH_4_]–and [M-H-C_6_H_6_O]^−^ respectively. In the MS/MS spectrum of [M−H]^−^ of BPAF, product ions at *m*/*z* 265 and 245 were assigned to [M-H-CHF_3_]^−^ and [M-H-CH_6_F_3_]^−^ respectively. In case of BADGE and its derivatives (BADGEs) MS/MS spectra of ammonium adducts and the most abundant or most characteristic product ions were recorded. BADGEs showed the characteristic division of the cleavage of the phenyl-alkyl bond with the simultaneous loss of the NH_3_. Moreover, two fragments ions were observed characteristic for two different ether chains regarding the hydrolyzed epoxy group.

BADGE•H_2_O showed an ion at *m*/*z* 209 corresponding to this fragmentation, and an ion at *m*/*z* 135, [C_9_H_11_O]^+^, which contained the epoxy group. Similar fragmentation was obtained for compounds which have two hydrolyzed or chloro-hydrozyled epoxy groups (BADGE•2H_2_O and BADGE•HCl). A common product ion at *m*/*z* 135 was observed due to the α-cleavage of the ether group.

Table 1 summarizes the two transitions selected for each compound and the optimum collision energies that maximize the intensity of the product ions, used for both quantitative and confirmatory purposes. The obtained data were confirmed from the MS/MS analysis of BPSs standards. The MRM mode acquisition was chosen for the quantification of BPSs in this study.

The linear range of studied bisphenols was ranging between 0.04 and 100 ng/mL. Validation parameters such as calibration data, including calibration equations, linearity presented as a correlation coefficient (R^2^) of the calibration curves, limits of detection (LOD) and quantification (LOQ), and precision (RSD) are presented in Table 2.

### 2.3. Application of the Procedure to the Real Samples

Amniotic fluid samples were obtained from 33 patients. Residues of bisphenols BPS, BPF, BPA, BPAF, BADGE•2H_2_O, BADGE•H_2_O•HCl, BADGE•2HCl, BADGE were detected in samples. Bisphenols BPE and BADGE•H_2_O were not identified in the analyzed samples.

**Figure 3:**
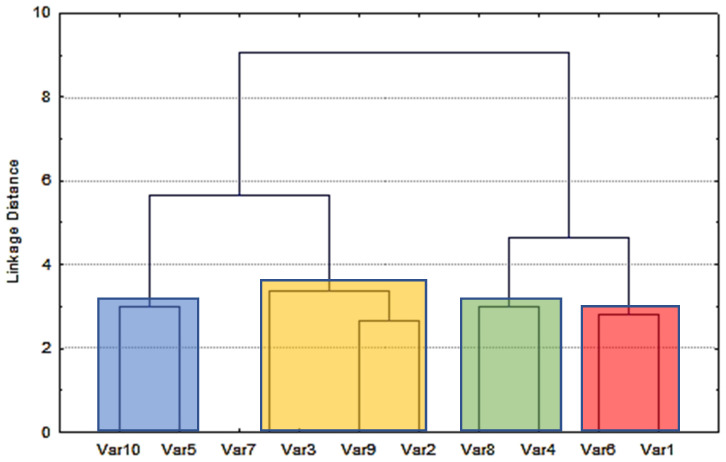
Dissimilarity dendrograms of the studied bisphenols related to the identification of them in amniotic fluid samples. BPS (Var1), BPF (Var2), BPE (Var3), BPA (Var4), BPAF (Var5), BADGE•2H_2_O (Var6), BADGE•H_2_O (Var7), BADGE•H_2_O•HCl (Var8), BADGE•2HCl (Var9), BADGE (Var10).

The results obtained of the identification in amniotic fluid samples were utilized for statistical evaluation. Ward’s method for data agglomeration and Euclidean distances for similarity measurements have been applied in the cluster analysis. The results presented in Figure 3 show that all studied bisphenols may be divided into four groups (marked with the four different colors), depending on the identification of them in real samples (containing 10 compounds listed in Table 1).

As shown in Figure 3, Ward’s method is a criterion applied in hierarchical cluster analysis. In the statistics, the nearest-neighbor chain algorithm can be used to find the same clustering defined by Ward’s method, in time proportional to the size of the input distance matrix and space linear in the number of points being clustered. At the initial step, all clusters are singletons (clusters containing a single point). The Ward’s method uses an analysis of variance approach to estimate the distance between clusters. The method aims at minimizing the sum of squares of any two (hypothetical) clusters that can be formed at each stage. To apply a recursive algorithm under this objective function, the initial distance between individual objects must be (proportional to) squared Euclidean distance. Ward’s method is considered very effective, although it tends to create clusters of small size.

Figure 3 shows the results, such as dissimilarity dendrograms that start at the bottom, where each bisphenol is its own cluster. As we move from bottom to top, bisphenols that are “close to each other” are clustered together. Each node in Figure 3 represents a connection of two or more clusters.

Looking at Figure 3 (indicated by blue, yellow, green and red colors) from bottom to top, it shows nodes that can be ranked from lowest to highest in the following order: 2.64 (BADGE•2HCl (Var9) and BPF (Var2)); 2.86 (BADGE•2H_2_O (Var6) and BPS (Var1)); 3.0 (BADGE (Var10) and BPAF (Var5)) and also (BADGE•H_2_O•HCl (Var8) and BPA (Var4)); 3.29 (BPE (Var3) and two bisphenols such as BADGE•2HCl (Var9) and BPF (Var2)); 4.71 (two pairs of bisphenols: BADGE•H_2_O•HCl (Var8) and BPA (Var4) and BADGE•2H_2_O (Var6) and BPS (Var1)) and the last node for values around 9.0 (which includes all bisphenols except BADGE•H_2_O (Var7)). Of course, for the highest value of “Linkage Distance” equal to 9.0, the greatest differences are observed. BPE (Var3) and BADGE•H_2_O (Var7) have not been identified in the real women’s amniotic fluid samples. Patients were classified on the basis of grouping the number of the detected compounds. However, this simple analysis caused us to apply more sophisticated methods of classification (presented in the Section 3).

## 3. Discussion

**Figure 4:**
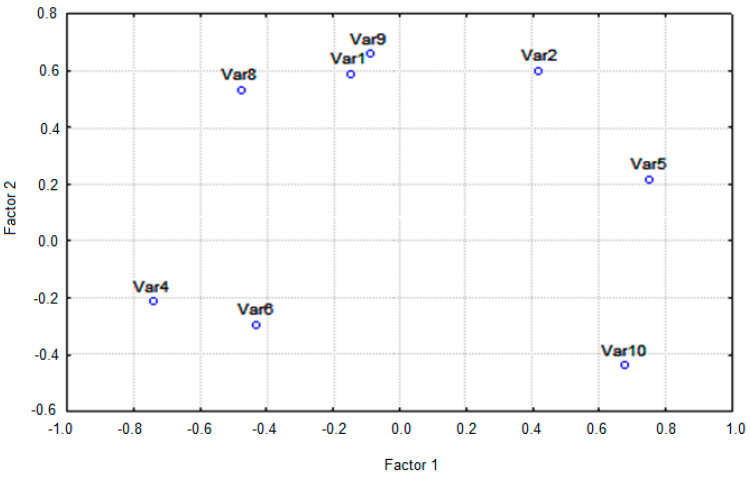
Factor scores of the analyzed amniotic fluid samples. BPS (Var1), BPF (Var2), BPE (Var3), BPA (Var4), BPAF (Var5), BADGE•2H_2_O (Var6), BADGE•H_2_O (Var7), BADGE•H_2_O•HCl (Var8), BADGE•2HCl (Var9), BADGE (Var10).

The same dataset was used to perform principal component analysis. According to the eigenvalue-one criterion, only the principal components (PCs) with eigenvalues greater than 1 are considered as the important one. The screen-plot shows that only two factors have fulfilled the criterion, based on the fact that the average eigenvalues of the autoscaled data is just 1. The cumulative explained variance for those PCs was equal to 98.1%. Figure 4 presents the score plot of all studied bisphenols in the space of the first two components (the week of pregnancy and frequency of the occurrence of bisphenols). Principal component loadings are presented in Figure 4 and correspond to the correlation coefficient of the particular variable. This figure indicates some groups of the identification of studied bisphenols in amniotic fluid samples.

BPE (Var3) and BADGE•H_2_O (Var7) are not shown in Figure 4 because these bisphenols have not been identified in the women’s amniotic fluid samples tested.

BPS (Var1) was detected in amniotic fluid samples collected from 16 patients (<LOQ) and was quantified in amniotic fluid samples collected from 12 patients. The highest concentrations of BPS were detected in samples of AF collected from two patients aged 44 and 39 years, at 15 and 16 weeks of pregnancy, respectively. Intermediate concentrations of this analyte have been detected in AF collected from patients aged 42, 39, 33, 24 years (at 16 and 17 weeks of pregnancy). BPS was less frequently detected in amniotic fluid samples collected from patients aged 29, 24, 26, 36 and 38 years (17 to 23 weeks of pregnancy).

BPF (Var2) has been identified in AF samples below the LOQ but in four of the oldest patients: P227–33 years (15 weeks of pregnancy; first pregnancy at increased risk of trisomy 18; test result: normal male karyotype); P245–39 years (15th week of pregnancy; missed miscarriage; impending fetal necrosis; risk of trisomy 21 (1: 301); test result normal male karyotype); P229–37 years (24th week of pregnancy; numerous fetal defects, including abnormal fetal structure, heart defect; the fetal karyotype had a polymorphism regarding the hetero chromatin block of chromosome pair 9 (9ph); no genomic imbalance was found in the DNA tested; test result diagnosed female chromosomal sex); P241–38 years (17th week of pregnancy; post-spontaneous abortion, uterine fibroid and perifallopian cysts; with increased risk of trisomy: trisomy 21 (1:45); trisomy 18 (1:253); test result normal male karyotype); P246–42 years (16 weeks of pregnancy; with increased risk of trisomy: trisomy 21 (1:142), test result normal female karyotype).

The highest amounts of BPA (Var4) were quantified in amniotic fluid samples from five patients aged 32 to 39 (15 to 18 weeks of pregnancy).

BADGE•2HCl (Var9) has been quantified in amniotic fluid samples from patients at the following ages (weeks of pregnancy in brackets): 44 (14), 28 (18), 30 (17), 36 (18).

BPAF (Var5) has been quantified in AF samples from the following patients: P8–29 years, P5–28 years, P229–37 years, P249–24 years, P222–33 years, P241–38 years, P246–42 years. In patient P8 an abnormal male karyotype was found, the presence of an additional chromosome 13 in the examined karyotype determines the occurrence of a number of features in the fetus that make up the clinical picture of Patau’s syndrome. In contrast, patient P249 had an abnormal female karyotype with the presence of a marker chromosome was diagnosed as an isochromosome derived from the *p* arms of the 5 chromosome, with a break point in the p10 band, in mosaic form. The tested DNA showed a genome imbalance in the form of duplication (partial trisomy) of the 5p short chromosome arm. The disorder described can be defined as a gene copy number change of a pathogenic nature not classified as a syndrome in Online Mendelian Inheritance in Man (OMIM). OMIM is a comprehensive, authoritative compendium of human genes and genetic phenotypes that is freely available and updated daily.

BADGE (Var10) was detected in amniotic fluid samples collected from three patients and quantified in amniotic fluid samples collected from 13 patients. The detected amounts of BADGE (Var10) were the highest in amniotic fluid samples collected from three patients aged 39 to 41 years (15 or 16 weeks of pregnancy).

BADGE•2H_2_O (Var6) was detected below LOQ in amniotic fluid samples collected from 11 patients and quantified in amniotic fluid samples collected from 10 patients aged (weeks of pregnancy in brackets): 36 (16), 44 (19), 26 (22), 29 (19), 24 (21), 33 (16), 36 (23), 38 (17), 39 (17) and 44 (15).

BADGE•H_2_O•HCl (Var8) was detected in amniotic fluid samples collected from nine patients and quantified in amniotic fluid samples collected from seven patients (e.g., quantified in amniotic fluid samples taken from the two oldest patients, aged 38 and 44, at 17 and 16 weeks of pregnancy, respectively).

**Figure 5:**
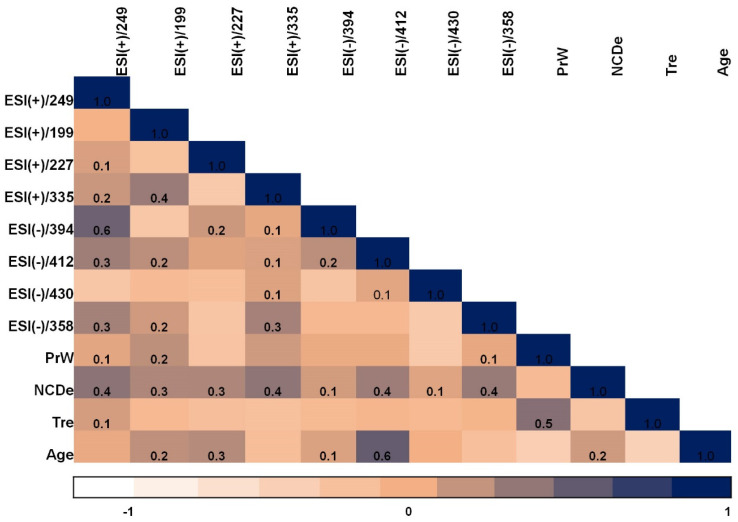
Pearson correlation matrix illustrating the significance between investigated variables. *PrW (pregnancy week), NCDe (the number of detected compounds), Tre (test results)*.

In order to run the correlation analysis for the investigated samples, parametric tests (Pearson correlation) were used. Pearson’s moment product correlation was used to highlight the similarities between patients, while as variables we considered the 33 investigated real samples. The Pearson test was chosen because it is a parametric statistical tool expecting a linear correlation between the investigated variables, suitable when those variables are coming from the same source, and/or they have the same measure unit. The correlation analysis is presented in Figure 5. The correlation matrix is shown presenting the level of significance (−1, 0, 1). Moreover, parametric tests revealed a generally strong positive correlation between studied bisphenols, when the number of detected compounds were assigned as variables.

In the case of the correlation between investigated variables, the pregnancy week (PrW), the number of detected compounds (NCDe), test results (Tre), age and the individual targets quantified (eight different bisphenols) of each sample were used. For the parameter Tre (test results), the test results determining the correctness of the fetal karyotype have been divided into the following five groups: first group–normal female karyotype; second group–normal male karyotype; third group–abnormal female karyotype; fourth group–abnormal male karyotype; fifth group–the correctness of the karyotype impossible to assess due to the failure to obtain a sufficient number of amniocytes during myotic division.

BPE and BADGE•H_2_O are not shown in Figure 5 because these bisphenols have not been identified in the women’s amniotic fluid samples tested.

Assessing the results presented in Figure 5, it can be seen that there is meaningful convergence: BADGE•H_2_O•HCl was identified in the samples of women older than average in the examined of group. There is also a correlation between the existence of two analytes in the samples: BADGE•2H_2_O and BPS. The number of detected compounds has been significant for the following analytes: BPS (ESI(+)/249), BPAF (ESI(+)/335), BADGE•H_2_O•HCl (ESI(−)/412) and BADGE (ESI(−)/358).

**Figure 6:**
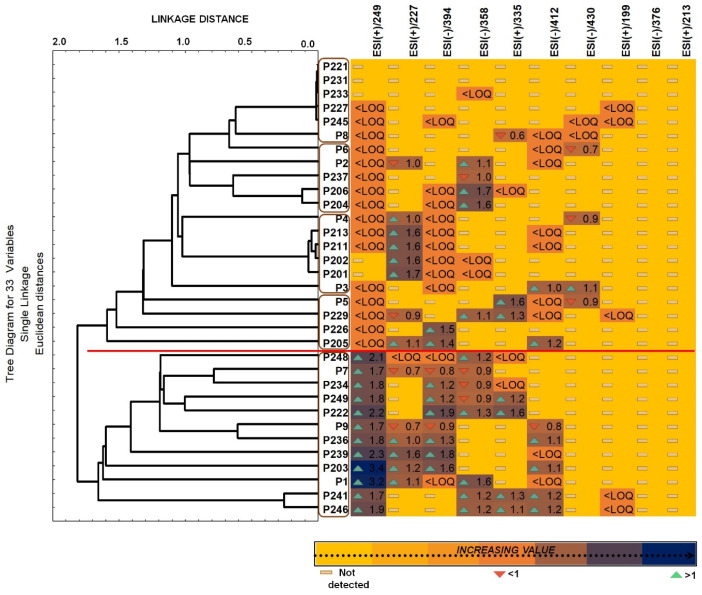
Heat map representing the quantity of investigated compounds detected in patient samples together with clustering analysis showing the correlation between patient samples; where *p* represents the number of the patient. <LOQ–below limit of quantification (in these cases bisphenols have been identified (but not quantified).

The correlation between the investigated variables is presented in Figure 6 in the form of a heat map combined with dendrograms of clusters’ analysis, which shows the formation of five main clusters. By the examination of the relationships between the mentioned groups (five patients’ groups), a strongly positively or negative correlation related to identification of the studied bisphenols in amniotic fluid samples. Hierarchical cluster analyses based on the amount of detected targets congregated the samples broadly based on the strength of the total amount. Hierarchical cluster analyses based on the amount of detected targets congregated the samples broadly based on the strength of the total amount. However, the dendrograms built with respect to the 33 variables selected, led to the formation of two main clusters with high significance, and three more with lower levels of significance.

In Figure 6, two main clusters with high significance can be distinguished, covering the results of samples taken from 33 patients: the upper one, which contains the results of samples taken from 21 patients, and the lower one, which contains the results of 12 patients. In the lower one, a significant amount of detected bisphenols is clearly visible, with values greater than 1.0 (darkest fields) for samples taken, among others, from patients P203, P1, P239, P222 and P248.

As was mentioned previously, for this data set we chose a parametric Pearson correlation, which presents a linear relationship between the two variables and is suitable to compare samples with various measurement units. BPE (*m*/*z* = 213) and BADGE•H_2_O (*m*/*z* = 376) were the only one variable which did not present correlation with others, and consequently it was removed from the matrix (they were not identified in real samples).

The heat map was built to express a snapshot of the quantified concentration of studied bisphenols in amniotic fluid samples. The real amounts are expressed in ng/mL. Regarding the fact that there is no information in the literature about the bisphenols content in the amniotic fluid studied in this work, so it is not possible to compare the obtained results.

In the present study, bisphenols were identified and quantified in samples from 33 patients. In the quantitative analysis, the concentrations ranges of bisphenols were as follows: for BPS from 1.68 to 3.38 (*n* = 12), for BPA from 0.69 ng/mL to 1.71 ng/mL (*n* = 14), bisphenol AF from 0.62 ng/mL to 1.63 ng/mL, BADGE•2H_2_O from 0.91 ng/mL to 1.91 ng/mL (*n* = 10), BADGE•H_2_O•HCl from 0.82 ng/mL to 1.23 ng/mL (*n* = 7), BADGE•2HCl from 0.68 ng/mL to 1.07 ng/mL (*n* = 4), and BADGE from 0.86 ng/mL to 1.74 ng/mL (*n* = 13). Bisphenols were also identified below their LOQs values (<LOQ) in 47 total samples: BPS (*n* = 16) BPF (*n* = 5), BPAF (*n* = 3), BADGE•2H_2_O (*n* = 11), BADGE•H_2_O•HCl (*n* = 9), and BADGE (*n* = 3).

The concentration of bisphenol residues in the AF may be influenced by endogenous factors and especially exogenous factors. The slightly correlations observed between the levels of the concentrations of bisphenols and also their frequencies in AF samples with chromosomal aberrations (confirmed cases of trisomy 21, 13 and 18 in some of the patients).

The highest concentration of BPS (3.38 ng/mL) and the concentrations of the following three bisphenols: BPA (1.24 ng/mL), BADGE•2H_2_O (1.61 ng/mL) and BADGE•H_2_O•HCl (1.13 ng/mL) were determined in the amniotic fluid sample of the patient’s No. 203. This fetus was diagnosed with: abnormal female karyotype; the presence of an extra chromosome 21, which determines the occurrence of Down’s syndrome in the fetus.

Additionally, concentrations of all four of the same bisphenols such as BPS (1.82 ng/mL), BPA (1.04 ng/mL), BADGE•2H_2_O (1.32 ng/mL) and BADGE•H_2_O•HCl (1.11 ng/mL) were also determined in sample No. 236. This fetus was diagnosed with Down’s syndrome. The tested DNA showed trisomy of chromosome 21 (21q11.2q22.3). However, the classical karyotype assessment was not possible because the metaphase plates necessary for microscopic analysis were not obtained.

The abnormal male karyotype was diagnosed in a different fetus: the presence of an additional chromosome 13 in the examined karyotype determines the occurrence of a number of features in the fetus that make up the clinical picture of Patau’s syndrome. The following concentrations of four bisphenols were identified in this collected amniotic fluid sample: BPS (1.74 ng/mL), BPA (0.69 ng/mL), BADGE•2H_2_O (0.84 ng/mL), and BADGE (0.86 ng/mL).

Additionally, the abnormal male karyotype was diagnosed in a different fetus: the presence of an additional chromosome 13 in the examined karyotype determines the occurrence of a number of features in the fetus that make up the clinical picture of Patau’s syndrome. In this amniotic fluid sample four bisphenols were identified such as BPAF (0.62 ng/mL) and three below their LOQs values: BPS, BADGE•H_2_O•HCl and BADGE•2HCl.

Another fetus was diagnosed with: abnormal female karyotype; the presence of an additional chromosome 18 determines the occurrence of Edwards’ syndrome. Three bisphenols’ concentrations were found in this AF sample (No. 205): BPA (1.06 ng/mL), BADGE•2H_2_O (1.42 ng/mL), BADGE•H_2_O•HCl (1.18 ng/mL) and BPS was identified.

Another abnormal female karyotype with the presence of a marker chromosome was also diagnosed as an isochromosome derived from the *p* arms of the 5 chromosome, with a break point in the p10 band, in mosaic form. The tested DNA showed a genome imbalance in the form of duplication (partial trisomy) of the 5p short chromosome arm in the region: 5p15.33p11, with a size of 46.11 Mb. The disorder described can be defined as a gene copy number change of a pathogenic nature not classified as a syndrome in OMIM. In the amniotic fluid sample (No. 249), relatively high concentrations of the following four bisphenols were determined: BPS (1.79 ng/mL), BPAF (1.17 ng/mL), BADGE•2H_2_O (1.22 ng/mL) and BADGE (0.86 ng/mL).

On the other hand, normal fetal karyotype (both female and male) was found despite the presence of relatively high concentrations of bisphenols in amniotic fluid samples (Nos. 222, 241, 246, 248, 1). In these cases, no genome imbalance was found in the tested DNA.

The mechanisms behind maternal-fetal transfer, and relationships between pregnant women and fetal exposures, remain unclear. The study of Zbucka-Krętowska assessed the impact of maternal exposure to BPA on the exposure of the fetus. Maternal plasma and amniotic fluid samples were collected and BPA was measured by GC-MS [38]. The median concentration of maternal plasma BPA was eight times higher than the total BPA concentration in the amniotic fluid (8.69 ng/mL, range: 4.3 ng/mL–55.3 ng/mL vs. median 1.03 ng/mL, range: 0.3 ng/mL–10.1 ng/mL) [38]. There was no direct relationship between the levels of BPA in maternal plasma and amniotic fluid levels. However, it negatively correlated with fetal development (birth weight) (R = −0.54, *p* < 0.001). The authors concluded that the risk of fetal BPA exposure depended on placental BPA permeability rather than the levels of maternal BPA plasma concentration [38].

Of course, the fetus is formed after conception and is formed in the prenatal period. The question that remains unanswered is whether the lifestyle of patients in the antenatal period and longer exposure to bisphenols may in any way affect the quality of the transferred genetic material and the abnormal formation of the fetus, trisomy or prematurity?

Although our thesis that bisphenols can affect fetal chromosomal abnormalities is controversial, we just want to justify the work in which in vitro studies have shown that BPA has cytotoxic and genotoxic properties, and may lead to chromosomal aberrations facilitating the predisposition of cells to neoplastic transformation [20].

Of course, the present study has some limitations. First, the study group consisted of a relatively small number of women with fetal chromosomal abnormalities. Second, the study group consisted of relatively small, generally number of patients. Unfortunately, the number of amniocentesis procedures has decreased, and recently thus far fewer patients have been examined. Further studies with a large sample size are needed in the future to confirm these relationships. Based on these preliminary results, the investigated relationships between the concentration of xenobiotics from bisphenols’ group in amniotic fluid samples can be recommended for further experiments in large-scale human biomonitoring studies.

## 4. Materials and Methods

### 4.1. Description of Patients Who Have Undergone Amniocentesis

The indication for amniocentesis was confirmation or exclusion of genetic aberrations, such as following trisomy: trisomy 13 (Patau’s syndrome), trisomy 18 (Edwards syndrome) and trisomy 21 (Down syndrome), and on heritable diseases in high-risk pregnancies, and also possibility of preterm birth [34].

The study group consisted of 33 pregnant Polish women aged between 24 and 44 years (in high-risk pregnancy, who were qualified for amniocentesis) [34].

The inclusion criteria for the amniocentesis included the following: abnormal ultrasound image of the fetus characteristic for trisomy 13, 18 and 21, especially revealing as heart defects, generalized swelling of the fetus, spina bifida, megacystis, brain defect, hydronephrosis, risk of infections as well as family history of trisomy 13, 18, 21 [34].

### 4.2. LC-MS/MS Analysis

LC-MS/MS analysis was performed on a triple quadrupole mass spectrometer (8050 Shimadzu (Kyoto, Japan)) equipped with LabSolution version 5.8 software for data collection and instrumental control. Electrospray ionization (ESI^−^/ESI^+^) was applied in the negative and positive ion modes. ESI-MS/MS spectrometer was coupled with UHPLC system (LC-30AD binary solvent delivery system, SIL-30AC autosampler and CTO-20AC thermostat) (Kyoto, Japan). The mass spectrometer was calibrated before the analysis using the manufacturer’s calibration solution. Both positive and negative ions were acquired and LabSolution software version 5.8 was used to control the LC/MS system and process data. Data were acquired in profile full scan mode (*m*/*z* 50–600). In positive mode [M+NH_4_]^+^ was selected as precursor ion, whereas in negative mode the precursor ion was [M–H]^−^. All of BADGEs tend to form adducts in positive ESI mode. Namely, ammonium adduct ions were obtained. In negative ESI mode, MeOH: water with no additives was used as a mobile phase to prevent ion suppression.

The ions were detected using a multiple reaction monitoring (MRM) mode. Positive and negative ions modes were both used to examine the compounds. The optimization of different MS parameters on the selectivity and MS response (MRM peak areas) for the studied compounds was carried out without a chromatographic column (Table 1). To select the MS/MS parameters, standard solutions of studied bisphenols at concentration 500 ng/mL were infused into the mass spectrometer using a Harvard syringe pump at a flow rate of 10 μL/min. The optimal parameters were as follows: interface temperature 300 °C, DL temperature 250 °C, heat block temperature 400 °C, nebulizing gas flow 2 L/min, drying gas flow 10 L/min, heating gas flow 10 L/min, interface voltage 3.5–4.5 kV, interface current 0.7 μA, and temperature of drying gas 290–350 °C. Nitrogen was used as the collision gas, and the collision energy used was 20–35 eV.

The analysis was carried out on a Kinetex C18 analytical column (100 mm × 2.1 mm, 1.7 µm) with column temperature set at 30 °C. The mobile phase consisted of water (mobile phase A) and methanol (mobile phase B) (for BPS, BPF, BPE, BPA, BPAF) and 40 mM ammonium formate in water and methanol (for BADGE•2H_2_O, BADGE•H_2_O, BADGE•H_2_O•HCl, BADGE•2HCl, BADGE). The mobile phase’s gradient program was as follows: 0–1 min, 25% B; 1–5 min, linear to 95% B; after that, returned to initial conditions at a flow rate of 0.4 mL/min. Stop and post-time were 7 min and 3 min, respectively. The injection volume was set to 1 µL. The autosampler’s temperature was set at 4 °C.

### 4.3. Validation Parameters

Standard solutions with known concentrations were prepared and analyzed to determine the areas corresponding to each concentration. To generate calibration curves, a minimum of six concentrations of each individual standard in fortified samples were measured. Calibration data, including calibration equations, linearity presented as a correlation coefficient (R^2^) of the calibration curves, limits of detection (LOD), limits of quantification (LOQ) and precision (RSD) are presented in Table 2. The linear range of studied bisphenols was ranging between 0.04 and 100 ng/mL. The limits of detection (LODs) and limits of quantification (LOQs) obtained for bisphenols were calculated according to the formulas LOD = 3.3 (SD/S), and LOQ = 10 (SD/S), where SD is the standard deviation of the response (peak area) and S is the slope of the calibration curve.

### 4.4. Statistical Analysis

To obtain results, they have been evaluated by cluster analysis (CA) and principal component analysis (PCA). CA and PCA were performed on a personal computer employing a Statistica package v.8.0 (StatSoft, Tulusa, OK, USA). The Ward’s method for data agglomeration and Euclidean distances for similarity measurements have been applied in the cluster analysis. Eigenvalue-one criterion was considered as the important ones for the principal component analysis. The data were further processed using Microsoft Excel. Microsoft Excel 2016 and Microsoft PowerPoint 2010 were used to prepare and integrate the figures composed of multiple parts.

## 5. Conclusions

In this study, a method based on SPE extraction combined with LC-MS/MS analysis was developed for the determination of 10 bisphenols in the amniotic fluid samples. Firstly, the SPE extraction conditions were optimized. Secondly, the combination of LC with ESI-MS/MS allowed for the rapid quantification of the selected compounds in real samples from the pregnant patients. High sensitivity is especially valuable for the evaluation of the studied bisphenols, which were present in the analyzed samples in small amounts. When searching into correlation based on investigated samples, we concluded that there was variability between investigated samples.

However, by obtaining all pools segregated in the same cluster with different levels of significance (five groups), we proved that they were representative for the given batch.

Amniotic fluid is an important diagnostic material, and it is commonly tested to detect chromosomal abnormalities, fetal anomalies and diseases. To date, only a few studies have investigated the relationship between bisphenols residues concentrations, or frequently present in AF samples collected during amniocentesis.

In the study, we present an impact of environmental bisphenols on fetal chromosomal abnormalities in Polish women by measuring concentrations of bisphenols in the amniotic fluid samples, illustrating a hypothesis suggesting that early bisphenol exposure of human fetus might induce chromosomal abnormalities. The present discussed the frequencies of chromosomal abnormalities and the concentrations of bisphenol residues in the amniotic fluid samples and suggest the hypothesis.

An attempt to explain the mechanism of individual influence of bisphenol residues on chromosome instability of human fetus, and consequently their influence to induce chromosomal abnormalities, could additionally confirm the correctness of our hypotheses; however, it will be possible after analyzing more amniotic fluid samples. Therefore, the availability of amniotic fluid samples is of great importance.

After interpreting the results, we can conclude that BADGE•H_2_O•HCl was identified in the samples of women older than average in the examined of group. The occurrence of two analytes together in the samples, BADGE•2H_2_O and BPS, is also correlated. The number of detected compounds has been significant for the following analytes: BPS, BPAF, BADGE•H_2_O•HCl and BADGE.

The highest concentration of BPS and the concentrations of the following three analytes: BPA, BADGE•2H_2_O and BADGE•H_2_O•HCl have been determined in the amniotic fluid samples. In both cases, the fetuses had an abnormal female karyotype, an extra chromosome 21, which is what causes Down’s syndrome in the fetus. The tested DNA showed trisomy of chromosome 21 (21q11.2q22.3). However, the classical karyotype assessment was not possible because the metaphase plates necessary for microscopic analysis were not obtained.

The abnormal male karyotypes were also diagnosed in a both different fetus with the clinical picture of Patau’s syndrome. The following analytes were identified in these collected amniotic fluid samples: BPS, BPA, BADGE•2H_2_O and BADGE; or BPS, BADGE•H_2_O•HCl and BADGE•2HCl.

Relatively high concentrations of the following four analytes: BPS, BPAF, BADGE•2H_2_O and BADGE have been determined in AF sample taken from a patient with another abnormal female karyotype (not classified as a syndrome in OMIM).

On the other hand, normal fetal karyotype (both female and male) was found despite the presence of relatively high concentrations of analytes in amniotic fluid samples. In these cases, no genome imbalance was found in the tested DNA.

Further testing of amniotic fluid samples will be needed for total bisphenols content and potential clinical risk.

Bisphenols are found in the natural environment and exposure to these xenobiotics negatively affects individual social groups, most often for many generations, which in turn may contribute to the development of specific disease entities in these human communities. It seems possible that there is an association between the concentrations of bisphenols residues in amniotic fluid samples collected during amniocentesis, and fetal abnormalities in pregnant women. We truly hope that our research preliminary results will initiate other teams of scientists to confirm or disprove the hypothesis described in the present study.

## Figures and Tables

**Table 1:** MS/MS conditions used for determination and identification of selected bisphenols in amniotic fluid samples.

Compound	Parent Ion, *m*/*z*	Quantifier ion [*Q1*], *m*/*z* Qualifier Ion [*Q3*], *m*/*z*	Collision Energy (eV); *Q1*, *Q3*	Drying Gas Temperature (°C)	Capillary Voltage (V)
[M−H]^−^					
**BPS** *Bis(4-hydroxyphenyl)sulfone*	249	108 156	22, 35	320	3500
**BPF** *4,4′-Methylenediphenol*	199	93 105	27, 24	320	4000
**BPE** *4-[1-(4-hydroxyphenyl)ethyl]phenol*	213	198 119	29, 35	320	4000
**BPA** *2,2-Bis(4-hydroxyphenyl)* *propane*	227	211 133	29, 30	290	4500
**BPAF** *4-[1,1,1,3,3,3-hexafluoro-2-(4-hydroxyphenyl)propan-2-yl]phenol*	335	265 245	31, 28	290	4500
**[M+NH_4_]^+^**					
**BADGE•2H_2_O** *2,2-Bis [4-(2,3-dihydroxypropoxy)pheny]* *propane* *Bisphenol A Bis(2,3-dihydroxypropyl) ether*	394	209 135	25, 29	320	3500
**BADGE•H_2_O** *3-[4-[2-[4-(oxiran-2-ylmethoxy)phenyl]propan-2-yl]phenoxy]propane-1,2-diol*	376	209 135	29, 31	320	3500
**BADGE•H_2_O•HCl** *3-[4-[2-[4-(3-chloro-2-hydroxypropoxy)phenyl]propan-2-yl]phenoxy]propane-1,2-diol*	412	227 191	32, 27	350	4000
**BADGE•2HCl** *2,2-Bis [4-(3-chloro-2-hydroxypropoxy)phenyl]propane* *Bisphenol A Bis(3-chloro-2-hydroxypropyl) ether*	430	227 135	26, 29	350	3500
**BADGE** *2,2-Bis[4-(glycidyloxy)* *phenyl]propane* *Bisphenol A diglycidyl ether*	358	191 161	30, 24	320	4500

**Table 2:** Calibration data of detected components including: calibration equations, linearity coefficient (R^2^), LOD, LOQ, and precision (RSD).

Compound	Regression Equation	R^2^	RSD%	LOD (ng/mL)	LOQ (ng/mL)
BPS	y = 15,061 + 5799	0.9998	2.42	0.27	0.81
BPF	y = 5993 + 348	0.9995	2.34	0.21	0.63
BPE	y = 7495 + 1740	0.9995	1.03	0.04	0.12
BPA	y = 9721 + 603	0.9993	2.15	0.06	0.18
BPAF	y = 4730 + 435	0.9990	1.90	0.06	0.18
BADGE•2H_2_O	y = 5025 + 511	0.9996	1.11	0.37	1.11
BADGE•H_2_O	y = 11,663 + 149	0.9996	0.93	0.28	0.84
BADGE•H_2_O•HCl	y = 4787 + 289	0.9993	1.46	0.21	0.63
BADGE•2HCl	y = 3816 + 869	0.9998	1.86	0.15	0.45
BADGE	y = 5043 + 308	0.9997	2.52	0.11	0.33

## Data Availability

Not applicable.
